# Neonatal Crohn’s disease with Oral ulcer as the first symptom caused by a compound heterozygote mutation in IL-10RA: a case report

**DOI:** 10.1186/s41065-019-0114-8

**Published:** 2019-12-26

**Authors:** Hongyan Lv, Baojun Qiao, Liyuan Fang, Lihong Yang, Qiuli Wang, Sujing Wu, Pengshun Ren, Lianxiang Li

**Affiliations:** 1 Department of Neonatology, Handan Maternal and Child Health Care Hospital, No. 6, Li Ming Street, Peace Road, Handan City, 056001 Hebei Province China; 2 Department of Neonatal Pathology, Handan Maternal and Child Health Care Hospital, No. 6, Li Ming Street, Peace Road, Handan City, 056001 Hebei Province China; 3 Department of Neural Development and Neural Pathology, Hebei University of Engineering School of Medicine, Handan, 056029 Hebei Province China

**Keywords:** Neonate, Oral ulcer, Crohn’s disease, Inflammatory bowel disease, Interleukin-10 receptor a, Genetic mutation

## Abstract

**Objective:**

To investigate the clinical and genetic characteristics of neonatal Crohn’s disease (CD), improve recognition of neonatal CD, and reduce the number of patients that are missed or misdiagnosed.

**Methods:**

A 10-day-old Chinese girl with oral ulcers was admitted to the Department of Neonatology. She later developed a rash and perianal disease, but without diarrhea and stool abnormalities. The patient and her parents underwent next-generation sequencing**.**

**Results:**

The results showed that the patient carries a compound heterozygous mutation in the interleukin-10 receptor A (IL-10RA) (NM_001558.3) gene. One heterozygous mutation was c.301 c > T, P. (Arg 101 Trp) in exon 3 of IL-10RA (a missense mutation), and the other was c. 537G > A, P. (Thr 179 =) in exon 4 of IL 10RA (a synonymous mutation). The patient’s father also carries the c.301 c > T, P. (Arg 101 Trp) heterozygous mutation in exon 3 of IL-10RA, whereas her mother carries the c.537G > A, P. (Thr 179 =) heterozygous mutation in exon 4 of IL-10RA.

**Conclusions:**

The results show that a compound heterozygous mutation in IL-10RA is associated with neonatal CD. Oral ulcers with a rash and perianal disease may be an early symptom of neonatal CD; therefore, such patients should undergo genetic identification as soon as possible.

## Introduction

Crohn’s disease (CD) is a subtype of inflammatory bowel disease (IBD); it is a chronic idiopathic inflammatory disorder of the gastrointestinal tract with unknown etiology. In CD, the inflammation is often transmural and may involve the whole gastrointestinal tract. Clinical manifestations include abdominal pain, diarrhea, intestinal granulomas, strictures, fistulas, perforation, fever, and malnutrition. The main pathological changes that occur during CD are thickening of the intestinal wall, non-cheese like granuloma nodules, and ulcers of varying depths on the mucosal surface. Depending on ethnicity, region, and age, the incidence of CD differs; the incidence rate in Europe is significantly higher than that in Asia. According to previous reports, the incidence of CD was found to be 0.848–2.7 per 100,000 [1–3]. CD often occurs in children and young adults, and the incidence rate is reported to be slightly higher in men than in women.

Neonatal CD is very rare. In the present study, we report the case of a 10-day-old girl with neonatal CD. The first symptom was oral ulcers. The patient later developed a rash, fever, perianal masses, and perianal pyoderma, without any clinical symptoms such as diarrhea, mucous stools, or bloody stools. As a result, we suspected an autoimmune disease; therefore, the patient and her parents underwent genetic sequencing. Finally, she was diagnosed with neonatal CD.

## Materials and methods

### Case presentation

A 10-day-old girl (Han Chinese) with oral ulcers was admitted to the Department of Neonatology in our hospital on July 22, 2018. After treatment, her condition did not improve; therefore, we suspected an autoimmune disease. This study was conducted in accordance with the Declaration of Helsinki and was approved by the Medical Ethics Committee of Handan Maternal and Child Health Care Hospital of Hebei Province. Written informed consent was provided by patient’s parents.

**Fig. 1:**
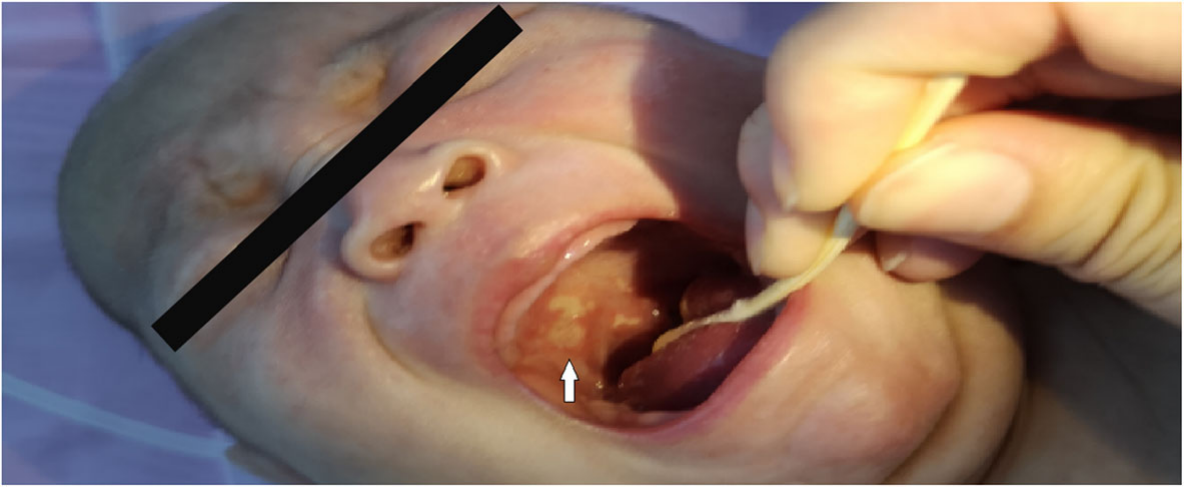
The patient had three ulcers on the mucosa of the upper palate and uvula. The surface of the ulcers is covered with a layer of white plaque (**arrow**)

**Fig. 2:**
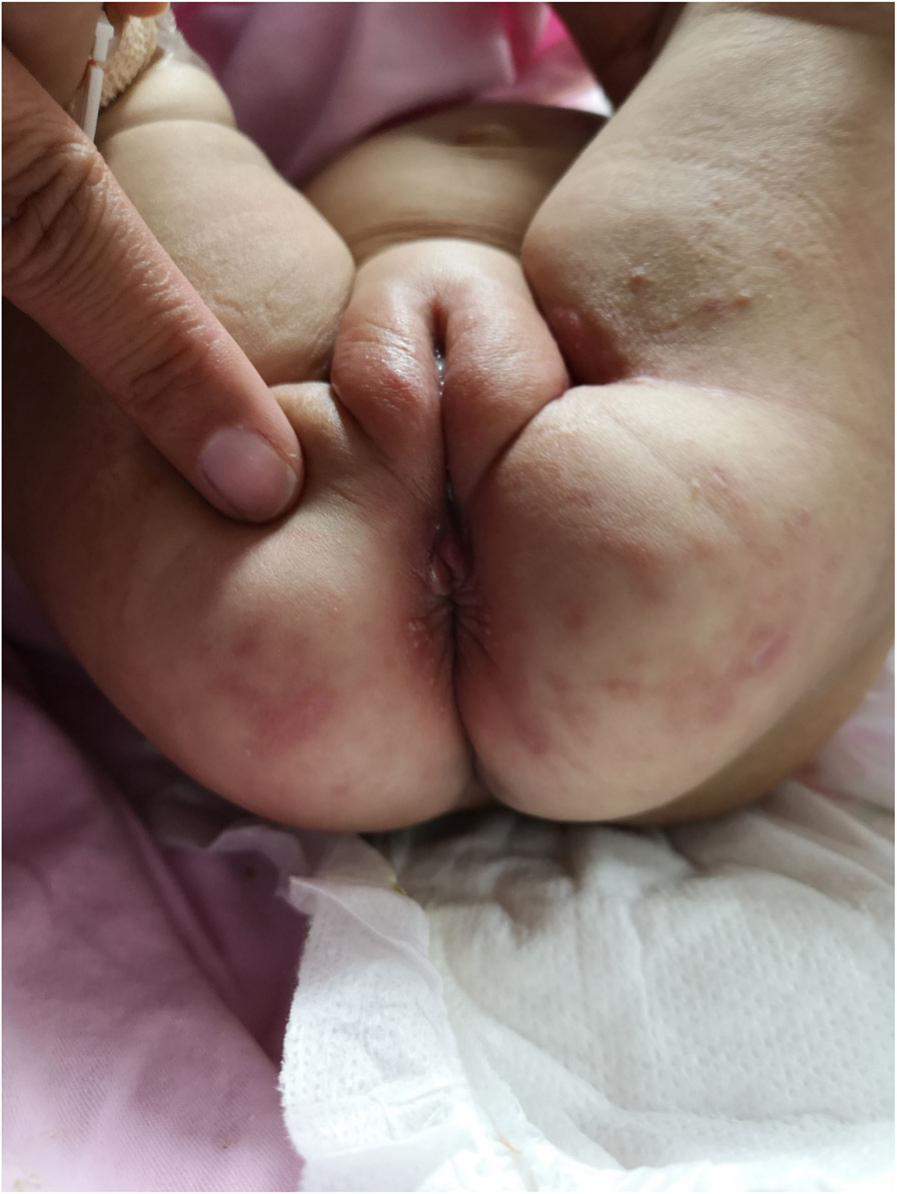
The protruding masses can be seen around the anus. In addition, a rash can be seen around the anus and inguinal region

Upon admission, the patient’s oral cavity was found to have many ulcers 10 days after birth. In addition, the external auditory canal had abnormal secretions. The shape of the oral ulcers were irregular, as was the size of the ulcers, and the surface was covered with a layer of white plaque that was not easy to wipe away **(**Fig. 1**)**. However, the patient had no clinical manifestations of diarrhea, vomiting, or bloody stools. Upon a physical examination, the patient was conscious and did not appear malnourished, had a body temperature 36.9 °C, heart rate of 136 beats minute, respiratory rate of 36 breaths per minute, blood pressure of 85/50 mmHg, and weighed 4.9 kg. There were three ulcers in the mucosa of the upper palate and the uvula had three ulcers, with varying shapes and sizes. The patient’s heart, lungs, and peristaltic waves were all normal. A neurological examination showed that the patient had no focal neurological impairments. After admission, she was initially diagnosed with neonatal oral ulcers and neonatal purulent otitis media. She received oral care, ofloxacin ear drops, amoxicillin, clavulanate potassium, antibiotics, and human immunoglobulin. On the fourth day after admission, she developed a fever with a red rash on her face, which was rice-like in appearance and pustule. The rash spread from her face to her armpits, chest area, groin, and perianal area. The laboratory results are shown **in** Table 1**.** The Erythra test was fungus positive and the fecal occult blood test was weakly positive. There were no abnormalities in coagulation factors. Therefore, she was treated with calamine lotion and fusidic acid cream, and with supportive care, the patient’s condition became slightly alleviated. However, 31 days after admission, she developed fever, oral ulcers, perianal pyoderma and perianal mass, and moderate anemia (Hb: 72 g/L) **(**Fig. 2**)**. The patient continued to be treated with calamine lotion, fusidic acid cream, antibiotics, and supportive care, and as a result, her symptoms were temporarily improved.

**Table 1:** Laboratory parameters

Laboratory index	Outcome	Reference value
Red blood cell count (RBC)	47 × 10^− 12^/L	4–5.2 × 10^−12^/L
White blood cell count (WBC)	22.4 × 10^− 9^/L	3.5–9.5 × 10^− 9^/L
Absolute value of neutrophils	9.46 × 10^− 9^/L	1.8–6.3 × 10^− 9^/L
Hemoglobin (HB)	70 g/L	110–160 g/L
C-reactive protein (CRP)	80.86 mg/L	0–5 mg/L
Erythrocyte sedimentation rate (ESR)	4 mm/h	0–20 mm/h
Complement hemolytic activity assay (CH50)	60.0CH50/ML	80–160.0/ML
Interferon-γ (IFN--γ)	35.53 pg/ml	0.00–2.10 pg/ml
Tumor necrosis factor (TNF)	57.84 pg/ml	130–8.55 pg/ml
Interleukin 10 (IL-10)	67.32 pg/ml	1.20–4.5 pg/ml
Interleukin 6 (IL-6)	115.40 pg/ml	0.00–4.55 pg/ml
Interleukin 4 (IL-4)	0.00 pg/ml	1.10–3.65 pg/ml
Total protein	51.8 g/L	62–83 g/L
Albumin	29.7 g/L	35–50 g/L
Globulin	22.1 g/L	20–40 g/L
Alanine aminotransferase (ALT)	123 U/L	9–52 U/L
Aspartate aminotransferase (AST)	113 U/L	14–36 U/L.

When taking a family history, we found that the patient’ brother developed fever, diarrhea, oral ulcer, and hyperbilirubinemia 5 days after birth, which developed in to septicemia and intestinal obstruction. Unfortunately, the patient’ brother died 30 days after birth and so was unable to provide corresponding genetic test results. According to the patient’s family history, we considered that the patient had an autoimmune disease and suggested that the patient and her parents should carry out genetic testing in order to obtain a definitive diagnosis.

### Next generation sequencing (NGS)

Genomic DNA was extracted from the patient’s and the patient’s parents peripheral blood. DNA was extracted from the blood sample using the QIAamp DNA extraction kit (QIAGEN NV, Hilden, the Netherlands), per the manufacturer’s instructions. The extracted DNA was purified using magnetic beads after DNA enzyme fragmentation. Next, the sample was PCR amplified and connected to the upper joint sequence. After capture and purification by TruSight One Sequencing Panel (Illumina Inc., USA) twice, the final library was obtained by PCR amplification and purification once again. The exonic regions of 4811 genes were sequenced by NextSeq500 Sequencing apparatus (Illumina Inc., USA). All data were compared to reference sequences using the BWA algorithm (UCSC hg19) by instrument default setting [4]. Data was annotated using literature reported methods [5]**.** After the screening process, bioinformatics software (PolyPhen2, LRT, Mutation Taste, et al.) was used to predict the results. The function, variation, and genetic pattern of each gene was analyzed, and suspicious candidate mutations were obtained. Amplification of suspected candidate mutation sites with PCR primers and Sanger sequencing, at the same time, enabled the corresponding loci of the patient’s parents to be detected.

### Literature search

In this study, information about CD was searched and collected from PubMed, CNKI, Wan Fang Date, and the Human Gene Mutation Database9 (HGMD). Searching terms included “neonate”, “Crohn’s disease”, “inflammatory bowel disease”, “interleukin-10 receptor gene (IL-10R)”, “gene mutation”, “oral ulcer”, and “perianal disease” as keywords. A list and comparison of 24 cases (including this case report) of IL-10R mutations were identified by genetic analyzing. The information and clinical features of the patients are shown in Table 2**.**

**Table 2:** Literature search results

Patients	Age/gender of onset	Mutation gene/type site/amino acid change	Clinical outcome	Parent gene	Author/ reference
1	30 d /female	IL-10RA / Homozygous c.537G > A / P.179 T > T	Clinical: Fever, diarrhea, bloody mucous stool, oral ulcers, anemia. ED: Scattered ulcers in colon and rectum. HD: Ulcers with chronic inflammatory cells and neutrophil infiltration. CRP: 71.4 mg/L; ESR: 58 mm/1 h	F: NR M: NR	Wang FP, et al. [2018] [6]
2	10 d /female	IL-10RA / Homozygous c.301C > T / P.101R > W	Clinical: Fever, diarrhea, mucous stool, rectovaginal fistula, anemia, innutrition, hypoproteinemia. ED: Multiple ulcers and polyp in ileocecum, colon, and rectum; segment presentation. HD: Acute and chronic inflammatory cell infiltration and granulation tissue. CRP: 22.0 mg/L; ESR: 11 mm/1 h	F: NR M: NR	Wang FP, et al. [2018] [6]
3	1 d /female	IL-10RA / Homozygous c.301C > T / P.101R > W	Clinical: Fever, diarrhea, perianal fistulas, anemia, innutrition, and hypoproteinemia. ED: Scattered ulcers in colon and rectum. HD: Acute and chronic inflammatory cell infiltration and mucosal erosive ulcer. CRP: 5.0 mg/L; ESR: 34 mm/1 h	F: NR M: NR	Wang FP, et al. [2018] [6]
4	Days /male	IL-10RB / Compound heterozygous mutation c. 301 C > T / P. 101R > w c.537 G > A / P.179 T > T	Clinical: Fistulas fever, diarrhea, perianal ulcer, anemia, innutrition, hypoproteinemia. ED: Irregular ulcers and false polyps in ileocecum, colon, and rectum. HD: Acute and chronic inflammatory cell infiltration with crypt inflammation and crypt abscess. CRP: 68.7 mg/L; ESR: 17 mm/1 h	F: NR M: NR	Wang FP, et al. [2018] [6]
5	1 d /male	IL-10RA / Compound heterozygous mutation c. 301 C > T / P. 101R > w c.350G > A / P. 117 R > H	Clinical: Fever, diarrhea, oral ulcer, anemia, perianal neoplasm, innutrition, hypoproteinemia. ED: Scattered ulcers in colon and rectum. HD: Acute and chronic inflammatory cell infiltration with ulcer and crypt abscess. CRP: 23.1 mg/L; ESR: ESR:29 mm/1 h	F: NR M: NR	Wang FP, et al. [2018] [6]
6	16 d /female	IL-10RA / Compound heterozygous mutation c. 301 C > T / P. 101R > w c.299 T > G / P. 100 V > G	Clinical: Fever, diarrhea, bloody purulent stool, perianal fistulas, anemia, innutrition, hypoproteinemia. ED: Scattered ulcers in colon and rectum. HD: Acute and chronic inflammatory cell infiltration with crypt inflammation and crypt abscess. CRP: 27.9 mg/L; ESR: 65 mm/1 h	F: NR M: NR	Wang FP, et al. [2018] [6]
7	NR /male	IL-10RA / Compound heterozygous mutation c. 537G > A / P. C 223 S c.668G > C	Clinical: Fever, diarrhea, bloody purulent stool, erythra in eye lid and back. ED: NR HD: NR. CRP: NR; ESR: NR	F: NR M: NR	Lu D, et al. [2015] [7]
8	NR /male	IL-10RA / Compound heterozygous mutation c. 537G > A / P. C 223 S c.668G > C	Clinical: Mucosanguineous feces, perianal abscesses, neoplasm. ED: NR HD: NR CRP: NR; ESR: NR	F: NR M: NR	Lu D, et al. [2015] [7]
9	4 d /female	IL-10RA / Compound heterozygous mutation c. 421G > A / P. 141G > W c.301C > T / P.101 R > W	Clinical: Fever, diarrhea, bloody stool, oral ulcers, anal fissure, hypoproteinemia, hepatosis ED: Extensive ulcers in colon, ileocecal erosion. HD: chronic inflammatory infiltration with crypt inflammation. CRP: +; ESR: -	F: NR M: NR	Jiang Y, et al. [2017] [8]
10	14 d /male	IL-10RA / Homozygous c.537 G > A / P.179 T > T	Clinical: Fever, diarrhea, bloody stool, oral ulcer. ED: Ulcers in colon. HD: Diffuse lymphocytic infiltration in ulcer. CRP: -; ESR: +	F: NR M: NR	Jiang Y, et al. [2017] [8]
11	10 d /female	IL-10RA / Compound heterozygous mutation c. 493C > T / P. 165R > X c.301C > T / P.101 R > W	Clinical: Fever, diarrhea, mucous bloody stool, anal fissure, Perianal lesion, erythra, hepatosis. ED: Extensive ulcers from colon ascending to the rectum. HD: chronic inflammatory with crypt inflammation and perianal neoplasm CRP: +; ESR: -	F: NR M: NR	Jiang Y, et al. [2017] [8]
12	14 d /female	IL-10RA / Compound heterozygous mutation c. 537G > A / P. 179 T > T c.301C > T / P.101 R > W	Clinical: Fever, diarrhea, bloody stool, oral ulcers, anal fissure, perianal fistulas. ED: Extensive ulcers in colon, ileocecal terminal erosion. HD: Acute and chronic inflammatory cell infiltration with crypt inflammation and crypt abscess. CRP: +; ESR: -	F: NR M: NR	Jiang Y, et al. [2017] [8]
13	9 d /male	IL-10RA / Compound heterozygous mutation c. 537G > A / P. 179 T > T c.301C > T / P.101 R > W	Clinical: Fever, diarrhea, bloody stool, oral ulcer, perianal neoplasm. ED: Ulcers in transverse colon. HD: Chronic inflammation of small and large intestine mucosa with crypt inflammation. CRP: +; ESR: -	F: NR M: NR	Jiang Y, et al. [2017] [8]
14	NR /female	IL-10RA / Compound heterozygous mutation c. 301C > T / P.R101 W C.350G > A / P.R 117 H	Clinical: Diarrhea, perianal fistulas. ED: NR. HD: NR. CRP: NR; ESR: NR	F: NR M: NR	Shim JO, et al. [2013] [9]
15	NR /male	IL-10RA / Compound heterozygous mutation c. 272A > G / P.Y91C C.784C > A / R 262 C	Clinical: Diarrhea, perianal fistulas, intestinal fistula. ED: NR. HD: NR. CRP: NR; ESR: NR	F: NR M: NR	Shim JO, et al. [2013] [9]
16	NR /female	IL-10RA/Compound heterozygous mutation c. 272A > G / P.Y91C C.301 C > T /.R 101 W	Clinical: Diarrhea, perianal fistulas, intestinal fistula. ED: NR. HD: NR. CRP: NR; ESR: NR	F: NR M: NR	Shim JO, et al. [2013] [9]
17	Neonate /male	IL-10RA / Homozygous c. 537G > A / P. 179 T > T c.301C > T / P.101 R > W	Clinical: Fever, diarrhea, bloody stool, anal fissure, perianal abscesses, skin infections. ED: Irregular ulcers and polyps in colon. HD: Inflammatory cell infiltration with ulcer. CRP: NR; ESR: NR	F: Homozygous C.737G > A M: NR	Fang YH, et al. [2018] [10]
18	4 months /female	IL-10RA / Homozygous c. 301C > T / P.R101W c.537G > A / PT179 T	Clinical: Fever, diarrhea, mucous bloody stool, perianal fistulas. ED: Many ulcers and a cobblestone-like vegetation in colon. HD: Inflammatory cell infiltration in ulcer. CRP: NR; ESR: NR	F: Homozygous C.737G > A M: Homozygous P > T179 T	Fang YH, et al. [2018] [10]
19	More than 1 month /female	IL-10RA / Homozygous C. 301C > T / P.R101W c. 470A > G / P.Y157C	Clinical: Diarrhea, mucous bloody stool, perianal fistulas, oral ulcer, innutrition, hypoevolutism. ED: Colon ulcers. HD: ND CRP: NR; ESR: NR	F: Homozygous C.301C > T M: Homozygous C.470A > G	Fang YH, et al. [2018] [10]
20	More than 1 month /male	IL-10RA / Homozygous C. 301C > T / P.R101W	Clinical: Bloody stool, erythra, repeated perianal abscess, innutrition. ED: Irregular ulcers and polyps in colon sigmoideum and rectum. HD: NR CRP: NR; ESR: NR	F: Homozygous C.301C > T M: NR	Fang YH, et al. [2018] [10]
21	Neonate /male	IL-10RA / Compound heterozygous mutation C. 301C > T / P.R101W C.350G > A / P.R117H	Clinical: Bloody stool, perianal fistulas, necrotizing enterocolitis, innutrition. ED: Many ulcers in rectum and polyps in colon. HD: ND. CRP: NR; ESR: NR	F: Compound heterozygous mutation C. 301C > T /P.R101W C.350G > A/P.R117H M: NR	Fang YH, et al. [2018] [10]
22	5 years 9 months /female	IL-10RB / heterozygous c.421G > A / P.141 K	Clinical: NR. ED: NR. HD: NR CRP: NR; ESR: NR	F: Heterozygous C.421G > A M: NR	Fang YH, et al. [2018] [10]
23	NR /male	IL-10RA / NR c.537G > A / P.T179 T	Clinical: Diarrhea, bloody stool, perinanal fistulas, oral ulcers, epifolliculitis. ED: Longitudinal ulcers in colon. HD: NR. CRP: NR; ESR: NR	F: NR M: NR	Yanagi T, et al. [2016] [11]
24	10 d /female	IL-10RA / Compound heterozygous mutation c. 301C > T / Arg101Frp c.537G > A / P.Tnr179 =	Clinical: Oral ulcers, fever, rash, perianal masses, perianal pyoderma, anemia, innutrition, hypoproteinemia, hepatosis. ED: ND. HD: ND CRP: 80.86 mg/L; ESR: 4 mm/1 h	F: Heterozygous C.301C > T/ Arg101Frp M: Heterozygous C.537G > A/ P.Tnr179 =	[This report]

*IBD* inflammatory bowel disease; *CD* Crohn’s disease; *NR* not recorded in detail; *ND* not detected; *ED* endoscope detection; *HD* histopathological detection; *CRP* C-reactive protein; *ESR* erythrocyte sedimentation rate; *F* father; *M* mother.; *NR* normal range

## Results

### Case findings

**Fig. 3:**
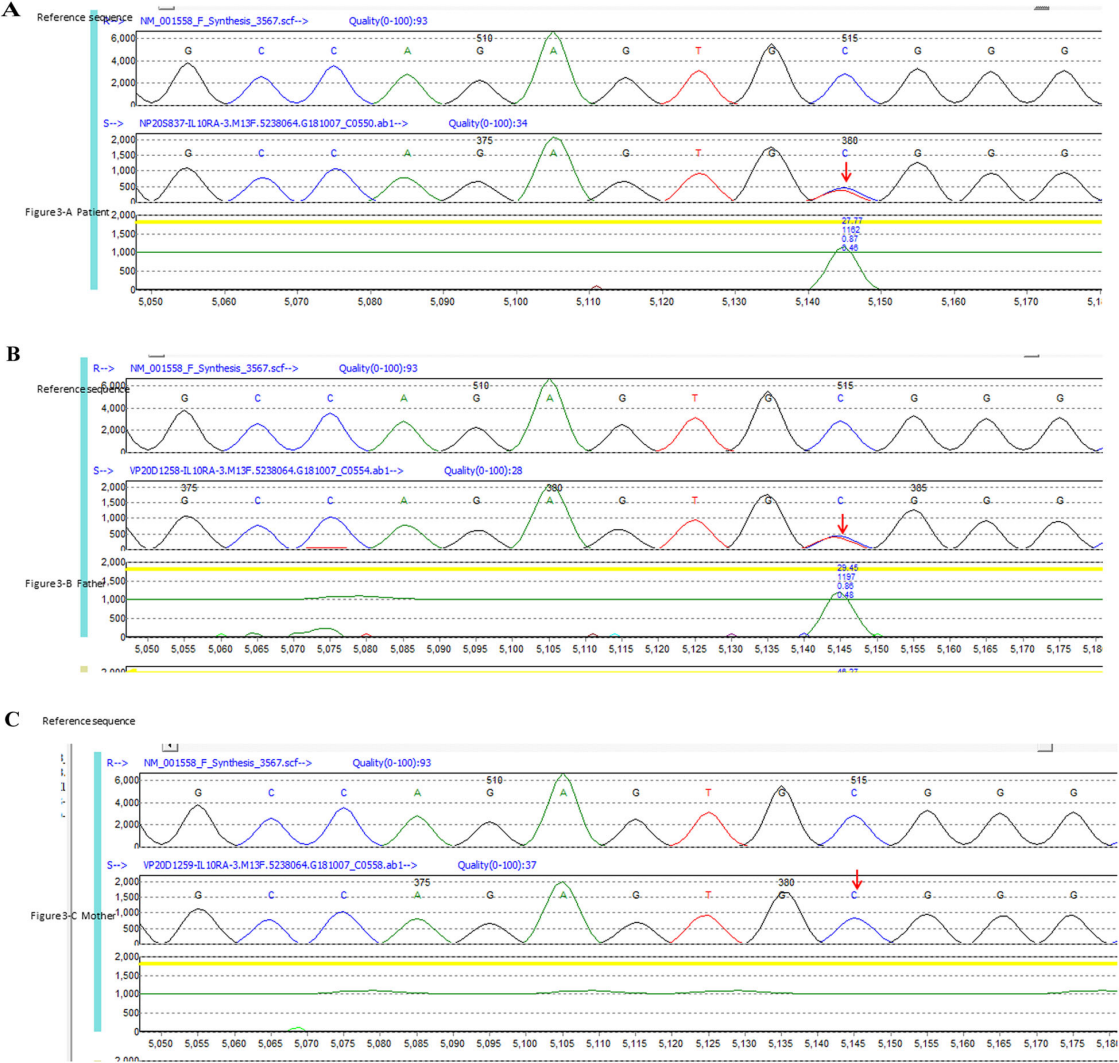
The patient carries one heterozygous mutation, c.301 c > T, P. (Arg 101 Trp), in exon 3 of the IL-10RA gene, which is a missense mutation (**a**, arrow). Her father also carries one heterozygous mutation, c.301 c > T, P. (Arg 101 Trp), in exon 3 of the IL-10RA gene (**b**, arrow). Her mother has no abnormalities in this coding region of the IL-10RA gene (**3-c**)

**Fig. 4:**
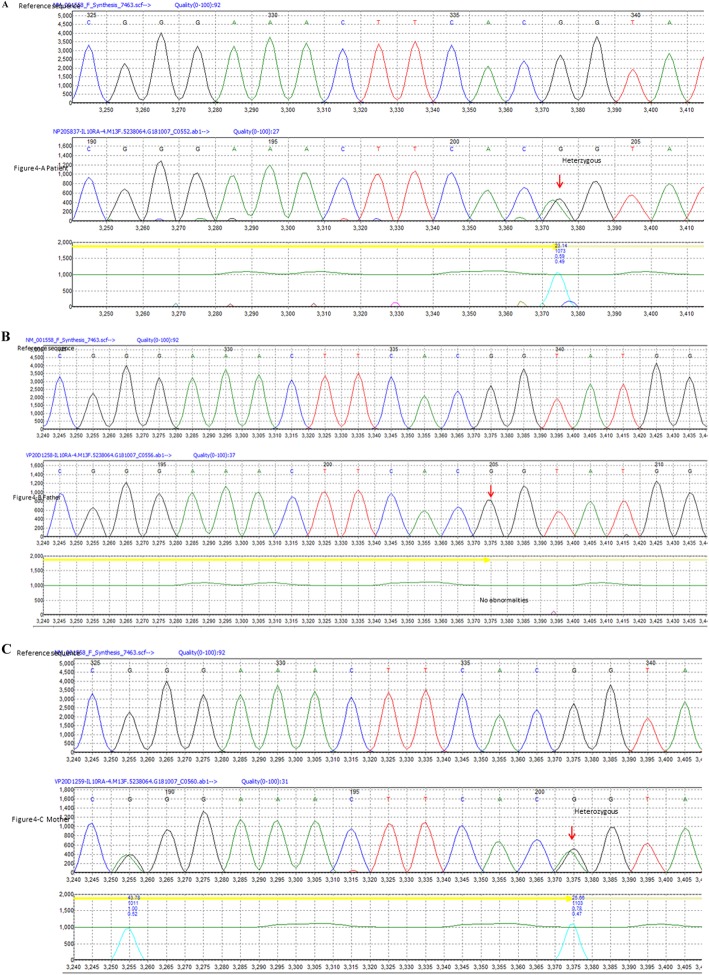
The patient carries one heterozygous mutation, c. 537G > A, P. (Thr 179 =), in exon 4 of the IL-10RA gene, which is a synonymous mutation (**a**, arrow**)**. Her father has no abnormalities in this coding region of the IL-10RA gene (**b**). However, her mother carries one heterozygous mutation, c.537G > A, P. (Thr 179 =), in exon 4 of the IL-10RA gene (**c**, arrow)

NGS identified a compound heterozygous mutation in the interleukin-10 receptor A (IL-10RA) (NM_001558.3) in this patient. One heterozygous mutation was c.301 c > T, P. (Arg 101 Trp) in exon 3 of the IL-10RA gene, which is a missense mutation (Fig. 3a). c.301 c > T, P. (Arg 101 Trp) has been previously reported as a suspicious pathogenicity mutation [12, 13], which results in the conversion of the 101st amino acid Arg to Trp. The second heterozygous mutation was c. 537G > A, P. (Thr 179 =) in exon 4 of the IL-10RA gene, which is a synonymous mutation (Fig. 4a). c. 537G > A, P. (Thr 179 =) has previously been reported as a pathogenicity mutation [13], and can affect mRNA splicing.

In order to investigate the genetic pattern, the patient’s parents also underwent NGS. The results showed that her father carries one heterozygous mutation: c.301 c > T, P. (Arg 101 Trp) in exon 3 of the IL-10RA gene **(**Fig. 3b). Furthermore, her mother also carries one heterozygous mutation: c.537G > A, P. (Thr 179 =) in exon 4 of the IL-10RA gene (Fig. 4c). The results showed that genetic mutations in the following genes were not present in the patient: XIAP, 33 NCF2 34, MEFV35, LRBA, XIAP, TNFR13B, and CYBB.

### Literature search findings

In addition, we collected information on 24 cases (including this case) from previous studies reporting on neonates with CD or early IBD caused by mutations in L-10 or L-10R. Their clinical manifestations and gene phenotypes are summarized in Table 2. The results show that the incidence rate of neonatal CD or IBD is equal regarding gender (except for one case with incomplete clinical records), fever occurred in 65.52% (15/23), diarrhea in 82.26% (19/23), mucous stools, bloody purulent stools, or bloody stools in 69.57% (16/23), oral ulcers in 39.13% (9/23), perianal diseases in 86.96% (20/23) (including perianal mass/neoplasms, perianal abscesses, perianal ulcer, perianal pyoderma, anal fistulas, anal fissure, and rectovaginal fistula), eczema/ rash in 21.74% (5 /23), anemia in 39.14% (9/23), hypoproteinemia in 26.09% (6/23), innutrition in 39.14% (9/23), liver dysfunction in 13.04% (3/23), intestinal fistula in 8.70% (2/23), intestinal necrosis in 8.70% (2/23), and folliculitis in 4.35% (1/23) of cases. In 24 cases of IL-10R mutations, IL-10RA mutations accounted for 91.67% (22/24) and IL-10RB mutations accounted for 8.33% (2/24). Regarding the gene mutation types (except for one case where it was not recorded), a compound heterozygous mutation accounted for 69.56% (16/23), a homozygous mutation accounted for 26.09% (6/23), and a heterozygous mutation accounted for 4.35% (1/23). The gene mutation site was c. 301C > T in 66.67% (16/24), c.537G > A in 41.67% (10/24), c.350G > A in 12.50% (3/24), c.272 A > G in 10.42% (2/24), c.688G > C in 10.42% (2/24), c.421G > A in 10.42% (2/24), c.493 C > T in 4.17% (1/24), c.737G > A in 4.17% (1/24), c.299 T > G in 4.17% (1/24), c.784C > T in 4.17% (1/24), and c.470A > G in 4.17% (1/24).

## Discussion

We report the case of a 10-day-old girl with neonatal CD diagnosed after genetic sequencing, which revealed a compound heterozygous mutation in the interleukin-10 receptor A (IL-10RA) (NM_001558.3) gene. One heterozygous mutation was c.301 c > T, P. (Arg 101 Trp) in exon 3 of IL-10RA (a missense mutation), and the other was c. 537G > A, P. (Thr 179 =) in exon 4 of IL 10RA (a synonymous mutation).

IBD includes three subtypes: ulcerative colitis, CD, and indeterminate colitis (IC). CD was first described by Crohn, Ginzterg, and Oppenheime in 1932. In IBD cases, CD is more common in children, but ulcerative colitis is more common in adults [14]. The prevalence of CD is 30–35% in children with very early inflammatory bowel disease (VIBD) [15].

Thus far, the etiology and mechanism of CD are not well understood. It is considered that CD is related to intestinal flora imbalance, immune system interactions, and genetic mutations [16]. Epidemiological investigations have shown that early intrauterine infection is associated with CD; in particular, the measles virus infection is an important risk factor [17]. However, other studies have failed to show an association [18]. In recent years, some scholars have found that the occurrence of CD is related to the birth season, as the risk of CD was reported to be higher for those born in the first half of the year [19]. In contrast, another study reported that the highest risk of CD is in those born in the second half of the year, not the first [20].

The NOD2/CARD15 gene was discovered as the first susceptible gene in CD. As a result, the relationship between gene variation and CD has attracted much attention. The proband concordance rate among monozygotic twins is 6.3% for ulcerative colitis and 58.3% for CD [21]. These results indicated that the incidence rate of monozygotic pairs in CD is significantly higher than that of dizygotic twins; therefore, it is suggested that heredity factors play an important role in the pathogenesis of CD. Many epidemiological studies have shown that the occurrence of CD is related to many susceptible genes. With the rapid development and clinical application of genetics technology, the accuracy of neonatal CD diagnosis has greatly improved. To date, the known susceptibility genes for neonatal CD or VIBD include XIAP, 33 NCF2 34, MEFV35, LRBA, IL10 5, IL-10RA, XIAP, TNFRF13B, CYBB [22], and ABCB1 gene mutations [23]. However, the mechanisms by which these gene mutations lead to neonatal CD or VIBD are not yet clear. It is believed that these susceptible genes are involved in maintaining epithelial barrier function, affecting the phagocytosis of monocytes and granulocytes, and can also affect the balance between the pro-inflammatory and anti-inflammatory response [24]. Glocker EO et al. were the first to discover that mutations in genes encoding the α-subunit (IL-10R1, encoding gene IL-10RA) and the β-subunit (IL-10R2, encoding gene IL-10RB) of the interleukin-10 (IL-10) receptor could induce VIBD development [25]. These results have since aroused great interest for many scholars. Further study has confirmed IL-10 and IL-10 receptor gene mutations to be related to the phenotype of severe perianal diseases and VIBD, particularly in infants [26, 27], and to be the main cause of neonatal CD and enterocolitis [6–10, 24, 28, 29]. In the present study, the patient was eventually diagnosed with neonatal CD caused by a compound heterozygous mutation of the IL-10RA gene. However, laboratory tests showed that the patient had elevated levels of interferon-γ, C-reactive protein, IL-6, and IL-10 (Table 1). Based on this data, we speculate that the compound heterozygous mutation of the IL-10RA gene can lead to a dysfunction in the immune system, which develops a series of clinical manifestations of CD.

According to the type of gene structure change, gene mutations can be divided into four categories: base substitution, frame shift mutation, deletion mutation, and insertion mutation. According to the change in genetic information, gene mutations can be classified into three types: synonymous mutation, missense mutation, and nonsense mutation. Kelsen JR et al. reported on heterozygous missense variants of IL10RA and unidentified variants of MSH5 and CD19 in early IBD in newborns [30]. Huang Z et al. found that C301C > T (p.R101RW) and c.537G > A (PT179 T) were the most common mutations of the IL-10RA gene, accounting for 88.1% of all neonatal CD patients [31]. Yanagi T et al. reported a new mutation (c. 537G > AgnpT179 T) in exon 4 of the IL-10RA gene, resulting in unique splicing aberration, with a lack of signaling of the IL-10 receptor; the patient also developed immune thrombocytopenic purpura and transient features mimicking juvenile myelomonocytic leukemia [11]. Our patient carried a compound heterozygous mutation in the IL-10 RA, one heterozygous mutation occurred (c.301 c > T, P. (Arg 101 Trp) in exon 3 of the IL-10RA gene, which is a missense mutation and results in the conversion of the 101st amino acid from Arg to Trp. The other occurred (c. 537G > A, P. (Thr 179 =) in exon 4 of IL-10 RA gene, which is a synonymous mutation and can affect mRNA splicing. Moreover, her father carried a heterozygous mutation (c.301 c > T, P. (Arg 101 Trp) in exon 3 of IL-10RA gene; and her mother carried a heterozygous mutation (c.537G > A, P. (Thr 179 =) in exon 4 of the IL-10RA gene. In other words, the patient had two mutations in the IL-10RA gene, one from her father and one from her mother. The results suggest that neonatal CD is a disease of hereditary IL-10RA gene deficiency, with an obvious genetic background. In order to exclude the effect of diet on neonatal CD, we paid attention to the feeding situation of the patient. The patient had been breast-fed from birth to hospitalization and did not eat any other dairy products. As a result, we can rule out the effect of environmental factors leading to neonatal CD in our patient. This study is of great significance to the classification of VIBD pathogenic factors and for the expanding the gene mutation spectrum of CD.

The IL-10R gene includes IL-10RA and IL-10RB. In order to improve the understanding of CD/IBD caused by IL-10R gene mutations among neonatal pediatricians, we analyzed the characteristics of neonatal CD/IBD caused by IL-10R gene mutations. The results showed that a mutation in the IL-10RA gene (91.67%) is more common than that in the IL-10RB gene (8.33%). Compound heterozygous mutations (69.56%) are also common; with the C. 301C > T mutation being the first and C. 537G > A (41.67%) being the second. Regarding clinical manifestation, diarrhea of unknown cause accounts for 82.26%, mucous stools, bloody purulent stools, or bloody stools accounts for 69.57%, and fever accounts for 65.52%. With these early signs, doctors can easily consider neonatal CD/IBD; however, other symptoms or signs are often ignored by doctors, especially oral ulcers and erythra. If the patient only presents with oral ulcers, rash, and perianal disease, without diarrhea and stool abnormalities, most doctors would not consider neonatal CD. We believe that this is the main reason for misdiagnosis and missed diagnosis of neonatal CD. Previous literature reports that the accuracy rate of fever, bleeding, and diarrhea in CD patients is 84.62% [32]. Clinically, oral lesions are rare in CD patients [33], accounting for only 4.8% of the patients [34]. Our patient’ first symptom was oral ulcers before beginning to develop a rash, fever, perianal mass, and perianal pyoderma; she did not have any symptoms of diarrhea or abnormal stools after admission. As a result, we did not perform endoscopy or intestinal. We instead suspected that the patient had an autoimmune disease, and NGS was performed to obtain a clear diagnosis. It is probable that our patient did not present with diarrhea and abnormal stools because CD was early onset; therefore, we can understand the particularity of this case. Our case suggests that newborns admitted to hospital with oral ulcers, fever, rashes, and perianal lesions should be closely monitored and genetically screened for neonatal CD, even if the patient has no symptoms of diarrhea and abnormal stools. This is important to make a clear diagnosis in the early stages of CD in newborns.

Patients with IL-10 or IL-10R gene deficiency often present with hematopoietic cell defects and immune regulation disorder, which can be dangerous for the patient. Thus far, there is no effective treatment for neonatal CD. According to the known of pathogenesis and previous literature reports, in addition to the use of conventional drugs and surgical intervention, immunosuppressive methods can be tried, such as anti-tumor necrosis factor [35, 36], anti-IL-12/23 [37, 38], IL-1β or hematopoietic stem cell transplantation, or gene therapy [39, 40]. According to the present literature and our research, neonatal CD is most often caused by a IL-10RA gene mutation. However, further study is required regarding the prevalence of genetic variations in different ethnicities and regions.

In conclusion, neonatal CD is very rare. Mutations in IL-10RA, resulting in gene deficiency, is the main factor for neonatal CD. The correlation between genotype and clinical manifestations requires further investigation.

## Data Availability

The datasets generated during and analyzed during the current study are available from the corresponding author on reasonable request.

## Abbreviations

CD: Crohn’s disease
IL-10RA: Interleukin-10 receptor A
